# Primary urethral realignment should be the preferred option for the initial management of posterior urethral injuries

**DOI:** 10.4103/0970-1591.65416

**Published:** 2010

**Authors:** R. P. Shrinivas, Deepak Dubey

**Affiliations:** Department of Urology, Manipal Institute of Nephrology and Urology, Manipal Hospital, Airport Road, Bangalore - 560 017, India

**Keywords:** Pelvic fracture, posterior urethral injury, primary urethral realignment

## Abstract

The initial management of posterior urethral injuries is controversial. Options of management include immediate surgical realignment, early realignment using minimally invasive techniques or simple suprapubic catheter (SPC) placement followed by delayed urethroplasty. The latter method has been preferred by most urologists but the last couple of decades have seen increasing reports of early urethral realignment which have provided better if not similar results as SPC placement. In this article a detailed analysis of studies involving primary realignment has been presented to reinforce the argument in favor of this approach.

## INTRODUCTION

There are essentially two options for the initial management of traumatic posterior urethral injury; suprapubic catheter (SPC) placement followed by definitive urethral stricture repair after three to six months or primary urethral realignment, immediate or early (1-15 days following injury).[1] Early realignment has significant benefits as this approach is associated with a 50% decrease in stricture formation and strictures that result are usually manageable by simpler techniques like urethrotomy and dilatation.[2] However, the optimum approach to the initial management of posterior urethral injury remains controversial. This controversy stems from initial reports emphasizing high rates of impotence and incontinence in patients managed by primary urethral realignment.[3] In a review of 19 studies, comparing early realignment versus suprapubic tube placement and delayed urethroplasty, Webster *et al*.,[4] reported an average rate of impotence of greater than 30% for early realignment in comparison with a 12-15% rate associated with delayed repair. A systematic analysis of these studies demonstrated a lack of clarity in the methods used for primary realignment. While in some studies the techniques were not highlighted, in others a variety of techniques in the form of sutured anastamosis and different types of catheter tractions were mentioned. McAninch[5] noted an incontinence rate of 30% after primary realignment if traction was used and proposed this to be a result of ischemia of the internal sphincter. In a further review, Koraitim[1] compared all studies between 1953-95 involving three techniques of management: primary realignment, initial SPC placement with delayed urethroplasty and primary suturing. They reported the rates of stricture, impotence and incontinence with SPC (97%, 19%, 4%), primary realignment (53%, 36%, 5%) and primary suturing (49%, 21%, 56%) respectively. However, in this analysis, most studies reported on primary realignment involving open techniques, including railroading (sound to sound and sound to finger) alignment. Also, the rates of impotence were erroneously reported as high in some series.

## MECHANISM OF INCONTINENCE AND IMPOTENCE FOLLOWING POSTERIOR URETHRAL INJURY: RESULT OF INITIAL INJURY OR TYPE OF INTERVENTION?

Till very recently the prevailing concept was that manipulation of the urethra at the time of injury jeopardizes potency by further trauma to the Nervi erigentes which are closely associated with the prostatic apex. Kotkin and Koch[6] questioned this assumption by comparing incontinence and impotence in two subsets of patients with posterior urethral injuries. In the first group surgical alignment was performed using railroading techniques and the second group had simple retrograde catheterization for partial or presumed complete urethral disruptions on retrograde urethrography. In both groups the postoperative potency rates were comparable (Group 1 -78% and Group 2- 70%). The authors concluded that their data ‘convincingly demonstrates that the injury itself and not the method of management is responsible for loss of potency and abnormal urination after urethral trauma’. There is a growing body of evidence that the injury itself results in loss of potency and abnormal micturition after urethral trauma. Using strict assessment criteria Shenfeld *et al*.,[7] demonstrated erectile dysfunction in 72% of patients with posterior urethral disruptions prior to urethroplasty. In a recent survey, Webster *et al*.,[8] interviewed 26 patients following posterior urethroplasty for pelvic fracture urethral distraction defects and found an impotence rate of 52%. Armenakas *et al*.,[9] studied 15 impotent patients before prostate membranous urethral reconstruction with pelvic magnetic resonance imaging (MRI) and duplex ultrasound, and showed that in 80% of cases the erectile dysfunction was vasculogenic and not neurogenic as was hypothesized. The most predictive signs of future erectile dysfunction on MRI were shown to be avulsion of the corpus cavernosum from the ischium, separation of the corporeal bodies, a displaced corporeal body fracture and lateral or superior displacement of the prostatic apex. Thus recent evidence confirms that it is not the type of initial management but rather the nature of injury itself that results in erectile dysfunction in posterior urethral injuries.

## IMMEDIATE SURGICAL URETHRAL REALIGNMENT WITH MINIMAL PARAVESCIAL DISSECTION

Immediate urethral realignment has been proposed to result in a much lower rate of subsequent stricture formation. Elliot and Barret[10] reported on 57 patients with traumatic posterior urethral disruptions managed with early surgical realignment with a mean follow-up of 10.5 years. Realignment was performed by an open technique using either Davis interlocking sounds or combined antegrade and retrograde catheterization, with minimal manipulation of the prostatic apex and retropubic hematoma. The procedure was successful in all patients. The urethral catheter was placed for a mean of 5.5 weeks (range 2 to 10). Postoperatively 66% patients developed strictures the majority of which were managed by occasional office dilatation and only 24% required some kind of urethral intervention. This study confirmed that primary urethral realignment is not associated with increased incidence of impotence and results in the formation of simpler strictures. Similarly, Asci *et al*.,[11] compared outcomes of posterior urethral injury using two techniques: immediate surgical urethral realignment (Group 1) and initial cystostomy (Group 2). In Group 1 45% and Group 2, 83.3% patients developed strictures. The incidence of impotence was similar in both groups [Group 1-17.6%, Group 2-20% (*P*=0.855)]. Mouraviev *et al*.,[2] compared outcomes of initial management of posterior urethral injury in two groups: 57 patients with early open realignment and 39 patients with initial SPC placement. Over a mean follow-up period of 8.8 years, the reported rates of urethral stricture, impotence and incontinence in the early realignment group were 49%, 34% and 18% and initial SPC were 100%, 42% and 35% respectively. All strictures were treated with one dilatation and direct visual internal urethrotomy (DVIU), which was successful in roughly half the patients in each group. However, patients with early realignment required an average of 1.6 procedures compared with 3.1 for delayed repair.

## MINIMALLY INVASIVE URETHRAL REALIGNMENT

Since the early 1990s, several investigators[12–14] have used minimally invasive endo-urological and fluoroscopy-guided techniques to establish primary realignment of the urethra 1-19 days after injury, in patients who are hemodynamically stable. This approach involves dilatation of the suprapubic tract along with antegrade and retrograde cystoscopy, with passage of a guidewire or ureteral catheter across the defect to facilitate urethral catheter insertion. Moudouini *et al*.,[14] described outcomes of 29 hemodynamically stable patients with posterior urethral injury managed with early endoscopic realignment. All patients were initially managed by a suprapubic tube and subsequently underwent endoscopic-guided realignment using both antegrade and retrograde approaches. Realignment was successful in 27/29 patients. Urethral strictures subsequently developed in 41% patients of which only three patients required extensive urethroplasty whilst the remaining were successfully managed with minimally invasive techniques. Only four patients reported impotence postoperatively. Hadjizacharia *et al*.,[15] compared outcomes of 21 patients with acute urethral injuries managed by immediate endoscopic realignment (IER) versus delayed treatment. IER was associated with a significantly low stricture rate (14%) versus delayed repair (100%).

Porter *et al*.,[16] used magnetic urethral catheters both retrogradely and antegradely to achieve alignment whereas Londergan *et al*.,[17] used multiplanar fluoroscopy using the Seldinger technique.

These studies strengthen the argument for immediate or early urethral realignment.

## THE ARGUMENT AGAINST INITIAL SPC PLACEMENT FOLLOWED BY DELAYED URETHROPLASTY

Initial placement of a SPC is associated with a prolonged period of catheter drainage (usually three to six months). Almost all patients managed by this technique will develop a pelvic fracture urethral distraction defect which requires extensive urethroplasty, the facility for which may be limited only to centers of excellence. Also there is a 10-12% failure rate associated with urethroplasty even in these centers.[18]

## CONCLUSIONS

In posterior urethral injuries due to pelvic fracture, impotence and incontinence are a result of the primary injury and not due to the type of initial management. Initial SPC placement is a safe technique but has an almost 100% incidence of stricture formation requiring extensive urethroplasty subsequently. With this approach patients have to wait three to six months for delayed repair and have to bear with the complications of long-term SPC placement. This approach may be used by surgeons inexperienced in primary alignment or where such facilities are unavailable.

In the stable patient with posterior urethral injury, primary urethral realignment, either surgical (with minimal paravesical dissection) or endoscopic, should be the treatment of choice since it results in a less than 50% incidence of stricture (most of which can be managed by minimally invasive techniques) and an acceptable rate of impotence and incontinence [Tables 1 and 2]. This decrease in the need for surgery has a large positive impact because the perineal approach anastomotic urethroplasty can be lengthy and arduous for the surgeon and the patient. If patients are being explored for concomitant intra-abdominal, bladder neck or rectal injury, an attempt at immediate primary realignment using railroading techniques (without disturbance of the retropubic hematoma) should be made. If there are no indications for surgical exploration, endoscopic realignment should be attempted at any time from 1-15 days after the patient has stabilized. These techniques should not be performed by inexperienced surgeons or in centers where facilities for endoscopy/fluoroscopy are not available. In experienced hands, such an approach has been demonstrated to have better outcomes as compared to insertion of a suprapubic cystostomy alone.[21]

**Table 1:** Recent studies with patients managed with immediate open surgical realignment and minimal paravesical dissection

Study	Number of patients	Stricture requiring open urethroplasty (%)	Impotence (%)	Incontinence (%)
Follis[19]	20	15	20	0
Elliot[10]	53	7.5	21	3.7
Kotkin[6]	15	6.6	24	12
Mouraviev[2]	57	24	34	18
Asci[11]	20	10	20	10
Patterson[21]	29	12	15	3

**Table 2:** Recent studies with early minimally invasive urethral realignment

Study	Number of patients	Stricture requiring open urethroplasty (%)	Impotence (%)	Incontinence (%)
Hadjizacharia[15]	14	0	NS	NS
Moudouini[14]	27	17	14	NS
Londergan[17]	5	0	40	0
Porter[16]	11	0	14	0
Jepson[20]	08	0	37.5	13

NS: Not stated.
